# Single-cell RNA sequencing reveals the immune microenvironment and signaling networks in cystitis glandularis

**DOI:** 10.3389/fimmu.2023.1083598

**Published:** 2023-02-06

**Authors:** Tai Lai Zhou, Heng Xin Chen, Yin Zhao Wang, Si Jie Wen, Ping Hong Dao, Yu Hang Wang, Min Feng Chen

**Affiliations:** Department of Urology, Xiangya Hosipital Central South University, Changsha, Hunan, China

**Keywords:** cystitis glandularis, urinary inflammation, urological diseases, scRNA-sequencing, immune microenvironment

## Abstract

**Introduction:**

Cystitis glandularis (CG) is a rare chronic bladder hyperplastic disease that mainly manifests by recurrent frequent urination, dysuria and gross hematuria. The current lack of unified diagnosis and treatment criteria makes it essential to comprehensively describe the inflammatory immune environment in CG research.

**Methods:**

Here, we performed scRNA-sequencing in CG patients for the first time, in which four inflamed tissues as well as three surrounding normal bladder mucosa tissues were included. Specifically, we isolated 18,869 cells to conduct bioinformatic analysis and performed immunofluorescence experiments.

**Results:**

Our genetic results demonstrate that CG does not have the classic chromosomal variation observed in bladder tumors, reveal the specific effects of TNF in KRT15 epithelial cells, and identify a new population of PIGR epithelial cells with high immunogenicity. In addition, we confirmed the activation difference of various kinds of T cells during chronic bladder inflammation and discovered a new group of CD27-Switch memory B cells expressing a variety of immunoglobulins.

**Discussion:**

CG was regarded as a rare disease and its basic study is still weak.Our study reveals, for the first time, the different kinds of cell subgroups in CG and provides the necessary basis for the clinical treatment of cystitis glandularis. Besides, our study significantly advances the research on cystitis glandularis at the cellular level and provides a theoretical basis for the future treatment of cystitis glandularis.

## 1 Introduction

Cystitis glandularis (CG) is a hyperplastic disease of the urinary tract epithelium that is caused by various chronic inflammatory stimuli (such as kidney stones, infection, and foreign bodies) and forms characteristic epithelial cell nests – known as Von Brunn’s nests – in the lamina propria (1, 2). In the past, cystitis glandularis was regarded as a rare disease. According to previous research statistics, the incidence of CG in all bladder diseases only accounts for 0.1%-1.9%, but with the development and wide application of endoscopy in clinical settings, as well as an enhanced health awareness in the general population, its diagnosis has increased in a yearly basis (3). Besides, there is still controversy in the diagnosis and treatment of CG, and most scholars believe that cystitis glandularis is a precancerous lesion that needs to be intervened in advance (4–6). However, some other experts believe that CG only needs regular follow-up, and current treatments’ curative effects are uncertain, which may increase the risk of treatment-related side effects (7). Therefore, until now, we still lack specific evidence that permits the implementation of unified management and treatment methods for CG.

In the G.DAVIES’ case report of 12 CG patients, the clinical manifestations of CG were associated with hematuria and recurrent urinary tract infection symptoms, and the pathogenic bacteria included *E.coli*, *klebsiella*, *Proteus*, etc (8). Meanwhile, LIU Xiaogang et al. successfully established a mouse model of CG using long-term *E.coli* stimulation, which confirmed that long-term lower urinary tract infection is closely related to CG (9). Thus, chronic urinary tract infection and inflammatory stimuli induce bladder epithelial cells to differentiate into more protective gland-like epithelial cells. This process forms columnar cells and the classic gland-like structure of Von Brunn’s nests, with mucus in the cytoplasm (10). Due to the similar pathological similarities between CG and bladder adenocarcinoma and the high incidence of coexistence of these two diseases, the correlation between CG and bladder adenocarcinoma has long been emphasized. But so far, there is still no accurate conclusion whether CG is a precancerous lesion of bladder adenocarcinoma (6, 11).

Previous reports have identified different types of inflammatory infiltration cells and the underlying mechanisms behind abnormal proliferation of urothelial cells in CG (12, 13). However, CG is formed by different kinds of epithelial, endothelial, and immune cells, that interact to gradually form the immune microenvironment of chronic bladder inflammation. The advent of scRNA-sequencing technology provides insights into the transcriptional and behavioral changes that occur at individual cells during disease development (14). This technique has been recently employed in cystitis, bladder and kidney cancer, as well as a variety of systemic urinary diseases, helping to clarify cellular composition and development trajectories of the immune microenvironment (15–17).

Our study performed single-cell RNA sequencing on inflammatory and surrounding normal tissues of different CG patients for the first time. It clears the characteristic of transcription, specific cell markers, and the cell-interaction of all kinds of cells, which provided substantial basis for further study of CG.

## 2 Material and methods

### 2.1 Acquisition of clinical samples

We included a total of 5 CG patients in this study with no previous history of bladder disease. Cystitis glandularis was confirmed by postoperative pathological diagnosis. Detailed patient information is available in **Supplementary Data**. All the inflammatory and surrounding normal tissues were obtained by transurethral resection. Due to the large tissue damage caused by transurethral cystotomy and the difficulty in obtaining bladder mucosa samples, the inflamed tissue sample of patient 324301 was excluded from the analysis due to poor data quality. Therefore, we obtained a total of 4 cystitis glandularis specimens and 3 surrounding normal tissue specimens after excluding some low-quality specimen data to conduct the following analysis. The whole experiment was supervised by the Ethics Committee of Xiangya Hospital of Central South University. The written consents from patients were obtained before sampling.

### 2.2 Preparation of single-cell suspension

The fresh human bladder tissue was immediately placed in GEXSCOPE^®^ tissue preservation solution (Singleron) after sampling. After this, the sample was removed from the preservation solution and cleaned in PBS (Hyclone, CAT.No.SA30256.01) for a total of 3 times, cut into smaller pieces, and placed in a centrifuge tube with 2 mL GEXSCOPE^®^ tissue dissociation solution (Singleron). The centrifuge tube was then placed at room temperature and shook for 15 minutes (180 rpm). After tissue dissociation, we added PBSA (containing 0.4%BSA) and filtered the cell suspension using a 400um sterile cell filter. Next, we centrifuged the filtered cell suspension (3000rpm, 5min, 4°C), poured the supernatant and added PBS for resuspension. A total of 2 ml of GEXSCOPETM erythrocyte lyser (Singleron) were added for 5 minutes, after which we added an equal volume of PBSA to terminate the reaction, centrifuged the cell suspension again, and added PBSA to clean and resuspend the cell precipitation. Finally, we used trypan blue standing to evaluate the cell activity under the microscope.

### 2.3 Droplet-based single-cell transcriptome sequencing

The bladder samples were diluted to 1x10^5 cells/mL, and the single-cell transcriptome library was prepared using Chromium Next GEM Single Cell 3′ Reagent Kits V3.1 (10x Genomics). According to Reagent Kits User Guide, we used the Chromium instrument for cell lysis and mRNA capture, then obtained cDNA solution by reverse transcription to construct the sequencing library. The amplified cDNA was used to construct the Illumina sequencing library. DNA sequencing was performed using an Illumina Novaseq 6000 sequencer.

### 2.4 Data preprocessing

**Figure 1:**
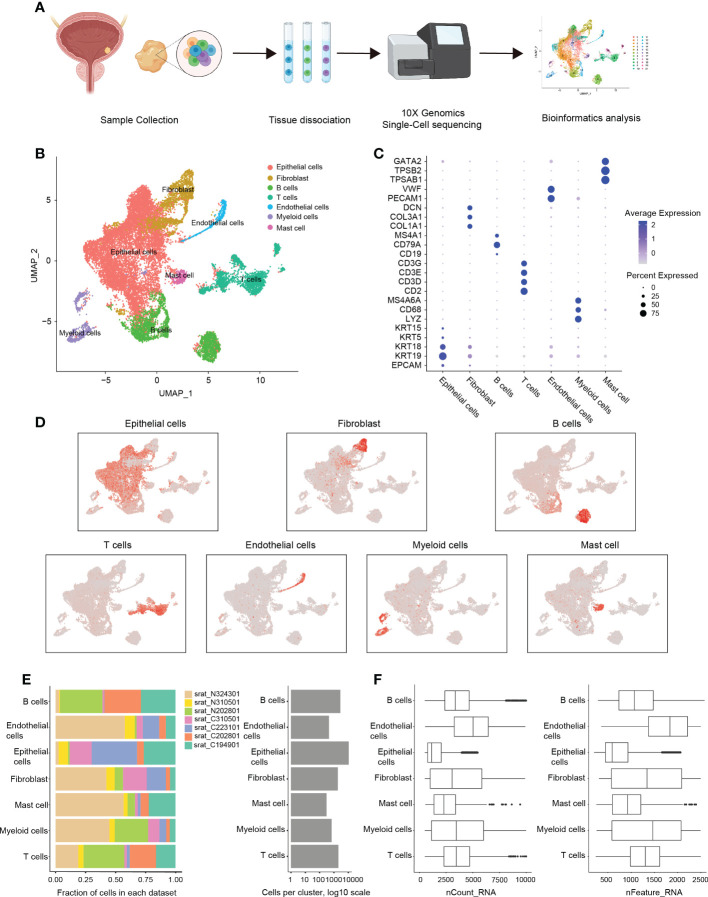
scRNA-sequencing analysis of the immune microenvironment in cystitis glandularis. **(A)** Workflow diagram of glandular cystitis sample collection and processing of scRNA-sequencing data. **(B)** Reduced dimension visualization U-MAP plot of scRNA-sequencing data obtained from all samples. Each color represents a cell type. **(C)** Dot plot of canonically expressed genes in each cell type after integration. **(D)** Distribution of canonically expression gene sets in each cell type present on the U-MAP plot. **(E)** Quantitative characteristics for each cell type. The samples starting with C are inflammatory tissues, and the samples starting with N are normal tissues. The number of cells per patient present in each cell type (left) and the total number of cells per cell type (right). **(F)** The detected gene expression value in each cell type (left) and featured number of genes (right).

We used CellRanger, a data processing software provided by 10X Genomics, to preprocess the single cell sequencing data in the fastq format. These data were compared to the human reference genome GRCh38 for cell and UMI counting to generate a gene expression matrix that contained a total of 32,851 cells and 23,428 genes. Moreover, we removed the possible doublets or low-quality cell populations following the cell cluster-based doublets removal method (for example, we excluded the low-quality cell groups in T cells – **Figure 1A**). The Seurat package was then used to read the cell-gene expressing matrix. In addition, we set the filtering criteria as follows: cells with nFeature_RNA > 200, nFeature_RNA < 2500, percent.Mt < 20 were included in further analysis. In addition, we excluded genes that were not expressed in at least 10 cells, as well as mitochondrial and ribosomal genes. This led to the inclusion of a total of 18,869 cells and 18,672 genes for further bioinformatics analysis.

### 2.5 Data integration and cell clustering

We used CCA (Canonical Correlation Analysis) of the Seurat package to remove and correct for batch effects among samples. After identifying anchors with the FindIntegrationAnchors function, we used the IntegratedData function to put the data together. Finally, we performed dimensionality reduction through PCA (principal component analysis), clustered the integrated data with the FindNeighbors and FindClusters functions, and showed cells in two dimensions using the UAMP method. Finally, the cell population was named according to previous classical cell markers (**Figure 1B**). In addition, the AddModuleScore function was used to score and visualize the classical markers of each cell type. In subsequent subpopulation analysis of each specific cell type, batch effects were removed in a similar fashion.

### 2.6 Identification of differentially expressed genes and GSVA enrichment analysis

The FindAllMarkers function was used to determine specific high expression levels of genes in each cell subset. In each subgroup, the ten genes with the largest avg_log2FC values were used as marker genes of each cell subset, and some markers may be highly expressed in more than one subset. We then used the GSVA (Gene Set Variation Analysis) package for unsupervised analysis of the defined cell subsets. We first obtained the human Hallmarker gene set (Species = “Homo sapiens”, category = “H”) using the msigdbr package, and then calculated GSVA enrichment scores for each subgroup using the gsva function. The pathway scores are normalized and presented as heat maps.

### 2.7 InferCNV analysis of epithelial cells

We used InferCNV to infer chromosomal copy variation between normal and CG tissue samples, with stromal cells as a standard reference. We visualized the gene variation on chromosomes and then calculated the sum of squares of inferCNV returned values of normal samples and CG tissue samples to compare the degree of potential gene changes in CG samples.

### 2.8 Cell trajectory analysis

Monocle2 was used to perform pseudo-time analysis of cell development trajectories of epithelial cells, T cells, B cells and myeloid cells. After the Seurat object was transformed into the CellDataSet object, the developmental differential genes (FDR<10%) were selected using unsupervised analysis. In addition, we used dimensionality reduction through the DDRTree algorithm on selected differential genes to construct cell development trajectories and two-latitude visualizations.

### 2.9 Scenic analysis

PyScenic was used to infer GRN (Gene Regulatory Network) in each cell subset and active transcription factors (TFs). We then used the PyScenic’s Grn, Cistarget and AUCell functions to score regulon activity of each cell subset and identify those with significantly higher subnetwork activity according to AUCell scores. Finally, a binary Regulon Binarized activity matrix was generated, and the final results were imported into the R software. The top 10 genes with the largest standard deviation among each cell subset were normalized and selected for heat map visualization.

### 2.10 CellChat analysis

We then analyzed cell-to-cell communication networks using CellChat, which evaluated receptor and ligand expression levels and inferred potential intercellular interactions. We projected gene expression data onto protein-protein interaction (PPI) networks to reduce dropout effects of signaling genes in single-cell data at shallow sequencing depths, and screened out signaling pathways expressed in less than 10 cells during analysis.

### 2.11 Multiplex immunofluorescence

The sections were dewaxed in xylene, absolute ethanol, and 85% alcohol in order. After washing with PBS, the tissue sections were heated in filled with EDTA antigen repair buffer (PH8.0) for 15 min for antigen repair. After 30 minutes of incubation with BSA, the primary antibody was added and incubated at 4°C overnight. On the second day, the slides were washed and the secondary antibody was added and incubated for 50 minutes. After incubation, fluorochrome was added to incubate at room temperature in the dark for 10 minutes, and the antigen repair and antibody incubation were performed again. After the tissue was covered with all fluorescent secondary antibody, the nuclei were counterstained with DAPI. The slides were placed under a scanner for image acquisition. (DAPI ultraviolet excitation wavelength 330-380nm, emission wavelength 420nm (blue light); The excitation wavelength of FITC is 465-495nm, and the emission wavelength is 515-555 nm (green light); CY3 excitation wavelength 510-560, emission wavelength 590nm (red light); IF647 excitation wavelength 656nm, emission wavelength 670nm (powder light); 620 excitation wavelength 590nm, emission wavelength 620nm (orange light). The antibodies used were COL1A1 (ABclonal, A1352), PECAM (ABclonal, A19014), PSTAT3 (ABclonal, AP0070) and IL6ST (ABclonal, A19014). A18036), PIGR (ABclonal, A61301), KRT19 (ABclonal, A19040), KRT15 (ABclonal, A4854), KRT13 (ABclonal, A0411), IL-2 (BIOSS, BS-1959R, CD8 (BIOSS, BS-10599R), FOXP3 (CST, 41822), P-STAT5 (BIOSS, BS-5703R), TNFR1A (ABclonal, A1540), NF-kB p65 (ABclonal, A11204), Vimentin (ABclonal, A19607), CD79a (ABclonal, A22452), CD27 (ABclonal, A11505), CD20 (ABclonal, A4893).

### 2.12 Statistical analysis

In this study, we used R (Version 4.1.0) to analyze the scRNA-sequencing data. All analytical methods, R packages and functions used can be found in the Material and Method section. We used the Student’s *t-test* for the comparison between the two groups and One-way ANOVA analysis to compare more than two groups. When P < 0.05, we considered the data to be statistically significant.

## 3 Results

### 3.1 Single-cell sequencing and cell cluster

**Figure 2:**
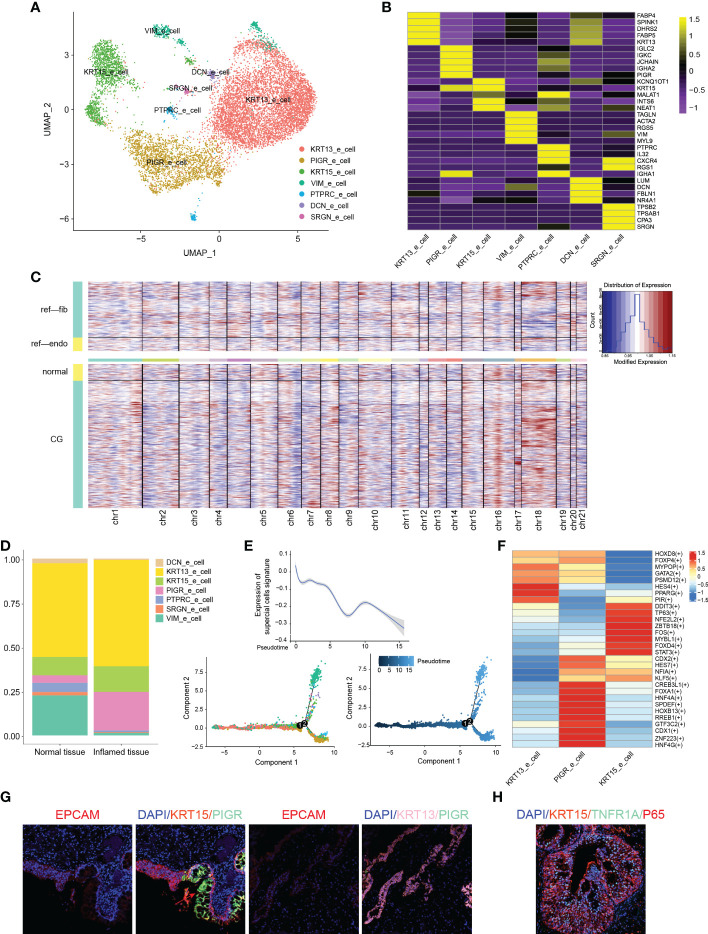
Classification and analysis of epithelial cells in cystitis glandularis. **(A)** U-MAP plot of different subsets of epithelial cells correlated by color. **(B)** Heat map showing Top5-specific genes expressed in epithelial cell subsets. **(C)** InferCNV was used to estimate the copy number variation score of epithelial cells, in which CG and normal bladder mucosa were compared to endothelial cells and fibroblasts. **(D)** The proportion of different kinds of epithelial subsets among all epithelial cells. The left panel indicates normal tissue and the right panel indicates inflamed tissue. **(E)** Pseudotime trajectory of epithelial cells of all samples inferred by Monocle2. Bottom: (left) pseudotime trajectory of epithelial cell subsets marked by color, and (right) the chronological order of development marked by color depth. Top: Change curve of the overall expression content of the superficial epithelial cell marker gene set based on the pseudotime trajectory of epithelial cell subsets. **(F)** Heat map showing the expression of major transcription factors in epithelial cell subsets using SCENIC. **(G)** Multiplex immunofluorescence verified various kinds of epithelial cell infiltration. **(H)** TNF-P65 inflammatory activation in KRT15 epithelial cells.

In this study, we included three CG patients with both healthy and inflamed tissue samples, and two separate inflamed tissue samples from other two CG patients. Among them, the inflammatory sample of patient 324301 will be excluded in the subsequent analysis due to factors such as excessive mitochondrial gene ratio. Therefore, a total of four CG samples and three normal bladder mucosa tissues located 5cm apart from the inflammatory area were acquired through transurethral resection. The pathological status was confirmed by HE staining and single-cell RNA sequencing was then performed using the 10× Genomics Chromium system (18) (**Supplementary Figure 1A**; **Supplementary Table S1**). After applying standard filtering criteria, we obtained a total of 18,869 cells (see Methods) on which we conducted bioinformatic analysis (**Figure 2A**). After merging and processing the data, we identified 21 cell clusters (**Supplementary Figure 1B**) that were further divided into seven types of cell groups (specifically epithelial cells, fibroblasts, B cells, T cells, endothelial cells, myeloid cells, and mast cells) following canonical cell markers (19) (**Figures 2B–D**; **Supplementary Figure 1C**; **Supplementary Table S2**). At the same time, after merging the data, cells from inflamed tissue and normal tissue were evenly distributed in each cell group (**Supplementary Figures 1D, E**).

We found that epithelial cells are significantly more numerous than other cell types, and significantly higher in CG than normal bladder mucosa (71.5% vs. 22.5%, P = 0.041), which is in accordance with the pathological characteristic of CG. We also observed that the proportion of endothelial cells in normal bladder mucosa was higher than in CG (1.1% vs. 5.4%, P = 0.032), which is consistent with previous scRNA-sequencing analysis of bladder tumors (20) (**Figure 2E**; **Supplementary Figure 2A**). Among immune cells, we found that the number of myeloid cells (including macrophages, monocytes, and DC cells) in normal bladder mucosa was significantly higher than in CG (2.3% vs. 8.3%, P = 0.034). At the same time, there is no significant difference in the proportion of B cells, T cells, fibroblast, and mast cells (**Figure 2E**; **Supplementary Figure 2A**). Besides, the endothelial cells had the highest transcriptional activity (**Figure 2F**).

### 3.2 The discovery of immune urothelium cells and the specific role of TNF

**Figure 3:**
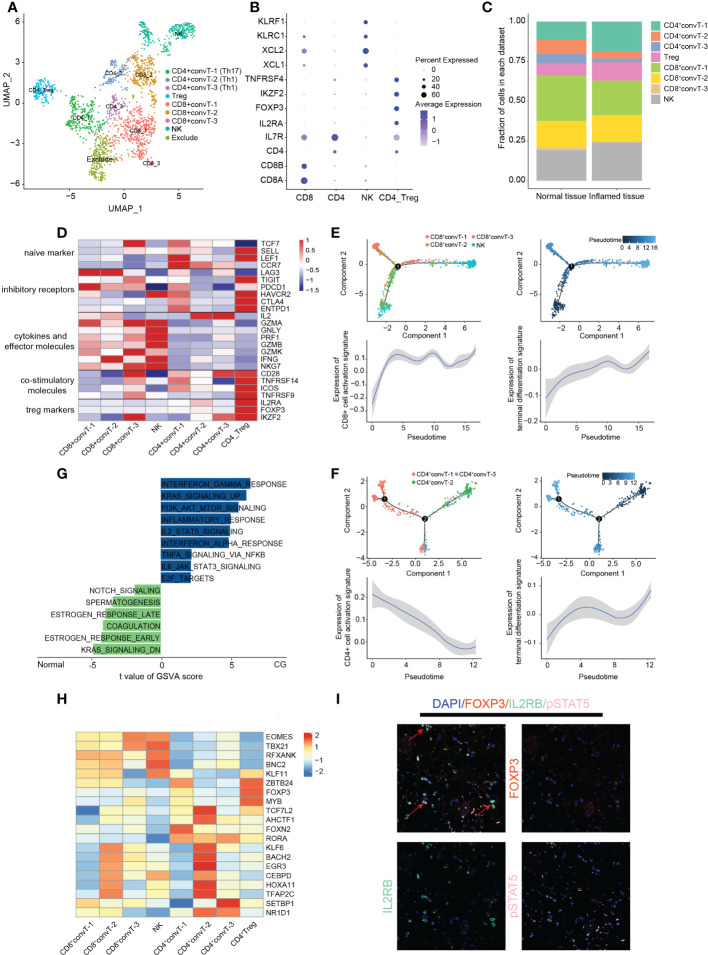
Classification and analysis of T cells in cystitis glandularis. **(A)** U-MAP plot showing different T cell subsets indicated by color. **(B)** Dot plot showing canonical genes used to distinguish different subsets of T cells. **(C)** The proportion of cells of different subsets of T cells in normal and CG tissues. The left panel indicates normal tissue and the right panel indicates inflamed tissue. **(D)** Heat map showing functional gene sets of different T-cell subsets. **(E, F)** Monocle2 inferences of the pseudotime trajectory of T cells. Above: pseudotime trajectory of CD4+ T cells/CD8+ T cells of all samples annotated with different colors or color depth, respectively. Bottom: Trends of expression of activated and terminated gene sets in CD4+ T cells/CD8+ T cells along with pseudotime trajectory. The gene sets used can be seen in supplementary table S5. **(G)** GSVA enrichment analysis of NK cells in CG and normal tissues. The horizontal axis shows the enrichment score, the right side shows the CG enrichment function, and the left side shows the normal tissue enrichment function. This figure displays only part of the Inflammation related pathway – see supplementary table S7 for all enrichment results. **(H)** Heat map showing the regulation of transcription factors within T cells. **(I)** Multiplex immunofluorescence showing the expression of the IL2RB-pSTAT5-FOXP3 activation pathway in Treg cells. The red arrow indicates the Treg cells activated by this activation pathway.

We further classified the 10,745 epithelial cells into seven subsets based on differential gene expression, and named each according to the most significantly expressed genes (**Figures 3A, B**; **Supplementary Table S3**). We then conducted inferCNV analysis in order to verify the possibility of CG transforming into bladder adenoma, but found no significant differences in chromosome copy number variation between CG and normal bladder mocusa (21). Moreover, CG did not display the classic chromosomal variation structure found in bladder tumors, such as 8q amplification, 8p deletion, and chromosome 9 variation (22) (**Figure 3C**; **Supplementary Figure 2B**). We also found that FAB (FAB4, FAB5), a fatty acid-associated binding protein mainly expressed in the intestine, was elevated in KRT13_epithelial cells, which indicated the possibility of intestinal-like metaplasia of CG (23). The epithelial cells of the female genital tract were once thought to be sentinels for antigen recognition, killing bacteria, and signaling activation of potential immune cells (24). In bladder mucosa, however, we discovered for the first time that the PIGR_epithelial cells have high expression levels of immunoglobulin-related genes, such as IGLC2, IGKC, IGHA2, JCHAIN, PIGR, etc. (**Supplementary Table S4**). Interestingly, Immunofluorescence histochemistry showed that PIGR epithelial cells mainly consisted of intestinal-type glandular cells (**Figure 3G**, red arrow). In addition, the proportion of PIGR_epithelial cells were significantly increased in CG (21.8% vs. 4.3%, P = 0.036) (**Figure 2D**; **Supplementary Figure 2D**), suggesting a critical role for PIGR_epithelial cells in immune defense and the activation of inflammatory response.

We then used Monocle cell trajectory analysis and found that PIGR_epithelial cells were mainly located at the end of the simulated cell differentiation trajectory, while KRT13_epithelial cells and KRT15_epithelial cells were found at the different sides at the beginning (25–27) (**Figure 3E**). Moreover, the expression of canonical markers of superficial epithelial cells decreased along the trajectory (**Supplementary Figure 2E**; **Supplementary Table S5**). Combined with the immunofluorescence results, we found that, in the differentiation process of urothelial cells into intestinal glandular cells, immunogenicity continues to increase and eventually leads to chronic bladder inflammation. At the same time, the expression of the EMT-related gene VIM, and the cytoskeletal proteins TPM2, CALD1, and ACTA2 gradually increased, highlighting the importance of these genes in the transformation of urothelial cells into intestinal-type cells (28, 29) (**Supplementary Figure 2F**).

Gene Set Variation Analysis (GSVA) showed that KRT13 epithelial cells highly expressed MYC gene-related proteins and fat metabolism-related genes (30). In recent years, research showed the great significance of the MYC oncogene to the differentiation and proliferation of many tumor cells and glycolysis metabolism (31). While PIGR_epithelial cells are enriched in protein secretion and IL-6 inflammatory pathway, KRT15 epithelial cells are enriched in inflammatory response and angiogenesis function, suggesting their essential role in the development of chronic inflammation (**Supplementary Figure 2G**).

**Figure 4:**
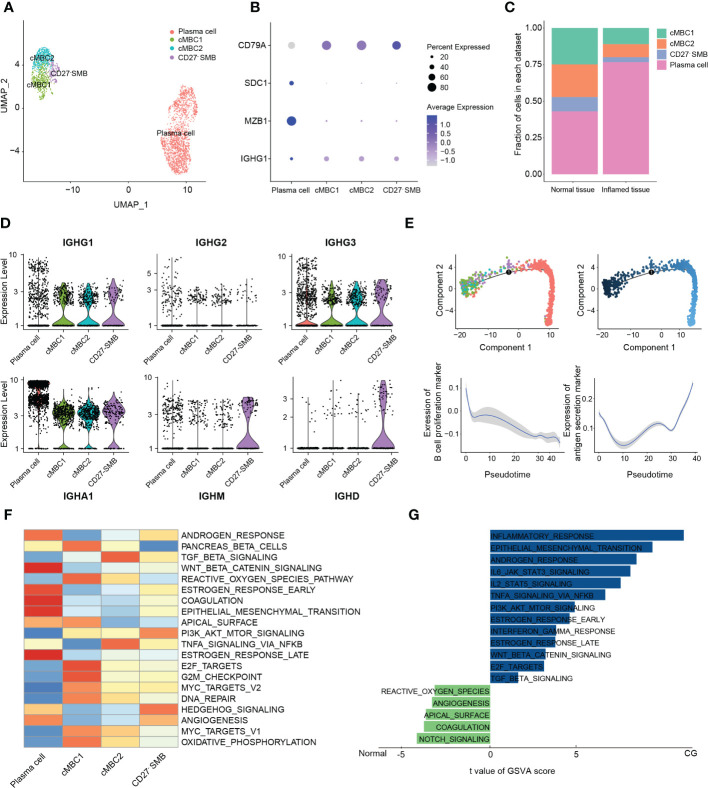
Classification and analysis of B cells in cystitis glandularis. **(A)** U-MAP showing the distribution of B cell subsets in different colors. **(B)** Dot plot used to represent the canonical genes of different B cell subsets. **(C)** The proportion of each B cell subset in CG and normal tissues. The left panel indicates normal tissue and the right panel indicates inflamed tissue. **(D)** Violin plot showing the expression levels of immunoglobulins in different B cell subsets. **(E)** Monocle2 showing the (top) pseudotime differentiation trajectory of B cells and (bottom) the change of proliferation and antigen secretion ability of B cells. **(F)** Heat map showing pathway activity of different B cell subsets using GSVA analysis. **(G)** GSVA calculation of the pathway activity of plasma cells between CG and normal tissues. The horizontal axis shows the pathway score, the right side shows the CG-enriched pathway, and the left side shows the normal tissue enriched pathway. This figure displays partial information only. See supplementary table 7 for further details.

SCENIC was then used to analyze related transcription factors and corresponding target genes of epithelial cells (32). This allowed us to uncover that the transcription factors TP63 and STAT3 are specifically expressed in KRT15 epithelial cells to regulate multiple cell growth factors and cyclins. Karin et al. showed that the activation of IL6 and TNF can lead to organ inflammation and activation of the oncogenic transcription factor STAT3 (33) (**Figure 3F**; **Supplementary Table S6**). We thus verified the unique effects of TNF on KRT15_epithelial cells using CellChat and immunofluorescence, and found it activates downstream NF-κB P65 pathway through the TNFRSF1A receptor to promote chronic inflammatory response and specific differentiation among epithelial cells (34) (**Figures 3H**, **4C**). Furthermore, we verified the existence of KRT15, KRT13, PIGR and VIM epithelial cells in CG (**Figure 2G**; **Supplementary Figure 2C**).

### 3.3 Changes of T cell activity in CG

We next divided the 2025 filtered T cells into ten clusters. Cluster-2 cells displayed a significantly higher mitochondrial and gene content than other groups, which were excluded in further analysis for the accuracy of data (**Supplementary Figures 3A, B**). According to the canonical markers of different types of T cells, we further divided T cells into CD8+convT cells, CD4+convT cells, Treg cells, and NK cells (**Figures 1A, B**). We discovered that except for CD4+convT-1 cells, there is no significant difference in the number of T cell subsets between inflamed and normal tissue in CG (**Figure 1C**; **Supplementary Figure 3C**).

After this, we compared the activation and suppression genes expressed in T cells and found that both CD8+convT-1 and CD8+convT-2 cells highly express the exhausted marker LAG3, with the former specifically expressing the inhibitory marker PDCD1. Meanwhile, CD8+convT-2 also highly secreted IFNG cytokines, which play an important role in differentiating T cells during inflammation. CD8+convT-3 cells had the lowest cell proportion and highly expressed a variety of cytotoxic factors, including GZMA, GZMK, or NKG7. As the main cytokine-secreting cell group, NK cells highly expressed a variety of cytotoxic factors except for IL-2, indicating its important role in the immune defense of CG (**Figure 1D**). Combining Monocle cell pseudotime trajectory with CD8+ T cell functional gene set expression curve, we found that CD8+convT-2 and some initial state NK cells gradually differentiated to end-stage exhausted CD8+convT-1 cells and activated NK cells, respectively (**Supplementary Table S5**). However, despite showing the strongest cytotoxicity, CD8+convT-3 cells were in the intermediate differentiation state with a small fraction in T cells (**Figure 1E**; **Supplementary Figure 3E**).

Among CD4 cells, Treg cells not only highly expressed naive markers and inhibitory receptors but also a variety of co-stimulatory molecules, which is consistent with previous results (19). At the same time, Treg cells also highly expressed TNFRSF family proteins (TNFRSF9, TNFRSF14), which play a crucial bidirectional role in the activating inflammation or suppressing T cell immune response signaling pathways. Moreover, we found that the CD4+convT-1 cells highly expressed the marker gene of Th17 (IL17A, IL17F, IL21), thus we classify CD4+convT-1 cells as Th17 cells. Besides, CD4+convT-2 and CD4+convT-3 highly expressed IL2, IL18R1 and CCR5, thus we consider that CD4+convT-2 and CD4+convT-3 are subpopulations of Th1 cells in different states (**Supplementary Figure 3D**). In addition to the naive markers LEF1 and CCR7, CD4+convT-1 cells also expressed a variety of cell depletion markers, such as HAVCR2, CTLA4, or PDCD1, suggesting a state of dysfunction. In addition, CD4+convT-2 and CD4+convT-3 co-expressed IL-2, an essential T cell growth factor that improves the activity of cytotoxic T cells and promotes T-cell secretion of a variety of cytotoxic factors, such as IFN-γ, TNF, and CSF (**Figure 1D**). According to Monocle pseudotime trajectory, we observed that the expression of various proinflammatory factors and chemokines decreased along the terminal differentiation of CD4+convT-2 and CD4+convT-3 cells into dysfunctional CD4+convT-1 cells (**Figure 1F**; **Supplementary Figure 1F**; **Supplementary Table S5**).Therefore, combined with the monocle results, we speculate that during the differentiation of Th1 cells to Th2 cells, T cells gradually transition to a dysfunctional state, suggesting that dysfunctional T cells may be one of the causes of long-term chronic bladder inflammation (35). According to GSVA enrichment analysis, the oxidative phosphorylation pathway was activated in Treg cells, demonstrating their highly active state in response to inflammation (**Supplementary Figure 3G**). Hence, by combining immunofluorescence and CellChat analyses, we found that Treg cells highly expressed IL2’s receptor IL2RB to activate the downstream pSTAT5 pathway and support the maintenance and expression of FOXP3, confirming the immune stabilization role of Treg cells in CG (**Figures 1I**, **4D**).

Based on the observed large proportion of NK cells in CG (23.90%) and their cytotoxicity effect on inflammation, we conducted GSVA enrichment analysis. Our results showed that, compared with normal bladder mucosa, NK cells in CG highly activate IL2, IL6, interferon, and E2F pathways, thereby activating the inflammatory and immune reactions (**Figure 1G**; **Supplementary Table S7**). SCENIC transcriptional analysis revealed that both NK cells and cytotoxic CD8+convT-3 cells expressed the transcription factors EOMES and TBX21, suggesting that these two transcription factors play an essential role in the regulation of immune defense (**Figure 1H**; **Supplementary Table S8**). According to previous reports, EOMES plays an important role in regulating cytotoxic factor secretion by CD8T cells and NK cells. The high expression of TBX21 also helps to induce CD8 cells into CX3CR1+ toxic cells (19).

### 3.4 Identification of CD27- switch memory B cell and immune activation of plasma cells in CG

**Figure 5:**
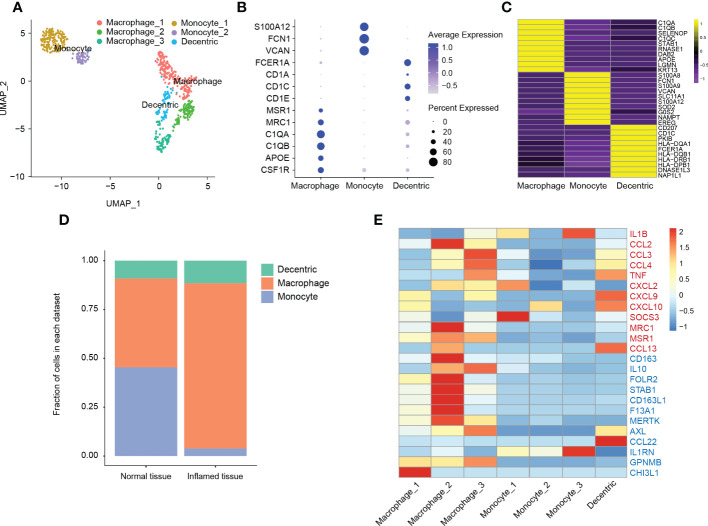
Classification and analysis of myeloid cells in cystitis glandularis. **(A)** U-MAP showing the dimensionality reduction distribution of different myeloid cell populations. **(B)** Dot plot showing the expression of classical genes of the myeloid cell subsets. **(C)** Heat map showing the top 10 differentially expressed genes in the myeloid cell population, ranging from yellow (high expression) to purple (low expression). **(D)** Cell proportion of different myeloid cell subsets in CG and normal tissues. The left panel indicates normal tissue and the right panel indicates inflamed tissue. **(E)** Heat map showing the genes associated with myeloid cell function. Red genes represent the M-1 type gene set (proinflammatory) and blue genes express the M-2 type gene set (anti-inflammatory).

B cells are key for adaptive immunity and play an important role in chronic infection through antigen presentation and immunoglobulin secretion. In this study, a total of 2747 B cells were divided into seven subsets (36) (**Supplementary Figure 4A**). Interestingly, cluster-5 cells expressed the immunoglobulins IGHA1, IGHG1, IGHM, IGHD, as well as a variety of memory B cell markers, such as CD45, CD83, or CD69. In contrast, we found low expression levels of the memory marker CD27, whereby we classified this cluster as CD27- switch memory B cells (CD27- SMB). Cluster-1 and -2 mainly secreted IGHG1, IGHG3, and IGHA mature immunoglobulins and numerous memory markers, whereby we defined them as conventional memory B cells (cMBC) (**Figures 5A, B, D**; **Supplementary Figure 4B**). Overall, we classified all B cells into conventional memory B cells-1 (cMBC1), conventional memory B cells-2 (cMBC2), CD27- SMB and plasma cells (20) (**Figure 5A**). Meanwhile, we demonstrated the existence of CD27- SMB in CG by multiple immunofluorescences (**Supplementary Figure 4C**).

We noticed that a large number of typical memory B cells differentiated into plasma cells in CG (76.8% vs. 43.1%, P = 0.044) (**Figure 5C**; **Supplementary Figure 4D**), which, as the final stage of B lymphocyte development, can secrete different types of antibodies and perform important humoral immune functions. While IGHA-type plasma cells were dominant in both normal bladder mucosa and CG, we found a significantly higher expression in CG. Moreover, the normal bladder mucosa is also mixed with part of IGHG3-type mature plasma cells (**Supplementary Figure 4E**).

Monocle pseudotime trajectory analysis showed that cMBC1 and cMBC2 were enriched at the beginning of the proliferation function, and that the proliferation ability of B cells decreased gradually during differentiation to plasma cells. Interestingly, the initial typical memory B and plasma cells conducted the antigen presentation function through different biological pathways (**Figure 5E**; **Supplementary Table S5**).

GSVA analysis showed that plasma cells were enriched in the estrogen receptor response pathway, which reportedly decreases the activation threshold of B cells and induces B cell maturation and growth (37) (**Figure 5F**). Since sIgA secreted by plasma cells is considered the first immune defense line on the mucosal surface, it can effectively inhibit pathogen adhesion, colonization, or invasion of the mucosal surface (38). Hence, we applied GSVA enrichment to show that plasma cells in CG were highly enriched with IL6, IL-2, E2F, and other inflammatory pathways, demonstrating the importance of B cells in the activation of CG inflammatory environment (**Figure 5G**; **Supplementary Table S7**). Moreover, IL-6 can also enhance IgA secretion and promote the division of IgA-type plasma cells to form a positive cycle of plasma cell proliferation (39).

### 3.5 Enrichment of immunosuppressive macrophages in CG

**Figure 6:**
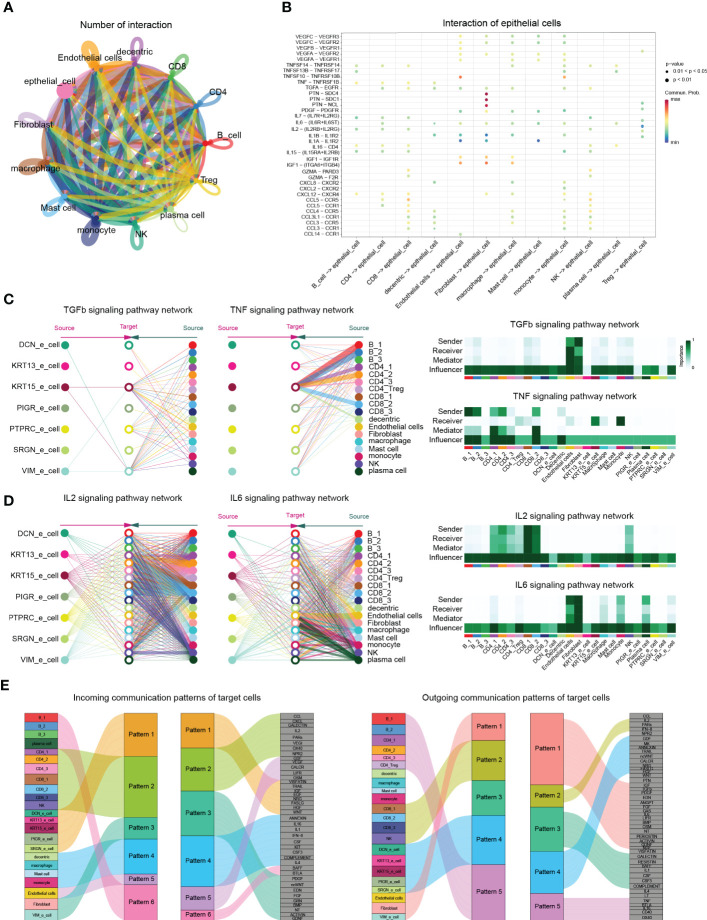
CellChat analysis postulates cell-cell interactions in cystitis glandularis. **(A)** The ability of cell communication between different cell types. Each cell type and their corresponding cellular signals are indicated by different colors. The arrows on the lines represent the direction in which cell signals are acting. The thickness of the lines represents the number of ligands and the intensity of cell signaling between different cell types. **(B)** Cell signals from different cell types to epithelial cells. The vertical axis shows the interaction between receptors and ligands in selected cell types, with different colors representing intensity. **(C, D)** Schematic diagram of the TGFb, TNF, IL2, and IL6 signaling pathways in each cell subset. (left) Stratified plots showing autocrine and paracrine effects of all cell subsets in each cell type. Each cell type is colored differently, and the thickness of the line segment indicates the strength of the cell effect. (right) Heat map showing the relative importance of all cell types in cell signaling pathways. According to the roles played by the different cells, they can be divided into sender, receiver, mediator and influencer cells. **(E)** Incoming (left) and outgoing (right) signaling patterns between secretory cells, demonstrating the relationships between various cell subsets with signaling pathways and hypothesized communication patterns. The size of each module represents the strength of the signal.

We continued to analyze 685 myeloid cells. According to classical myeloid cell markers, we divided these cells into macrophages, monocytes, and dendritic cells (**Figures 6A, B**). Compared to other cells, we found that DCs highly expressed a variety of human leukocyte antigens, demonstrating their role in antigen presentation of humoral immunity (**Figure 6C**; **Supplementary Table S9**). Among these leucocytes, we found a significantly higher proportion of macrophages (45.56% vs 84.62%, P = 0.021), and a significantly lower number of monocytes in CG (**Figure 6D**, P = 0.037). In addition, dendritic cells also expressed some typical monocyte gene markers, which suggested monocytes differentiate into macrophages and dendritic cells during chronic bladder inflammation (**Figure 6C**).

According to the functional gene set, macrophages are generally grouped into pro-inflammatory type M1 macrophages and anti-inflammatory type M2 macrophages (40). In this study, not only macrophage_1 (accounts for 54.8%) overexpressed CHI3L1, but also macrophage_2 (accounts for 25.5%) overexpressed a variety of M2-type genes, such as CD163, STAB1, or IL-10. In addition to the expression of M2-type genes, macrophage_3 (accounts for 19.9%) also expressed kinds of inflammatory chemokines, such as CCL3, CCL4, or TNF, which indicates an intermediate state between M1 and M2 differentiation. This suggests that macrophages are more likely to be in an M2-type immunosuppressive state during chronic bladder inflammation (41, 42) (**Figure 6E**).

### 3.6 Cell interactions in the CG chronic inflammatory environment

We used CellChat to evaluate the potential signaling pathways and interactions between different cell groups during chronic bladder inflammation (43). Interestingly, the intercellular communication between CD8 cells and NK cells is most closely, while epithelial cells are mainly affected by PTN, MK, PARs, IGF, TRAIL, EGF, as well as other cell proliferation and inflammation-related pathways (**Figures 4A, B**).

Since cystitis glandularis is an epithelial proliferative inflammatory disease, we continued to explore the effects of other cell groups on epithelial cells. According to CellChat, the proinflammatory pathway TGFβ is thought to be emitted by various immune cells and stromal cells, mainly acting on KRT15_epithelial cells. CellChat analysis showed that endothelial cells and fibroblasts act as primary transmitters and regulators of TNF in the immune microenvironment of CG, acting together on the TNFRSF1A receptor to activate NF-κB P65 downstream pathway in KRT15 epithelial cells (**Figures 3H**, **4C**; **Supplementary Figure 4F**). This also suggests the effectiveness of TNF-α inhibitors such as adalimumab, Infliximab, Certolizumab Pegol in treating CG.

At the same time, T cells and NK cells act as major sources of receptors and ligands in the classical IL2 inflammatory pathway and induce Treg cell activation through IL2RB (**Figures 1I**, **4D**; **Supplementary Figure 4G**). In the IL6 pathway, stromal cells, monocytes, and plasma cells promote endothelial activation through the IL6ST-PSTAT3 pathway (**Figure 4D**; **Supplementary Figures 5H, I**). These results at the single-cell level demonstrate that multiple classical inflammatory pathways play unique activation roles in different cell populations during chronic bladder inflammation.

Finally, we used the nonnegative matrix pattern recognition approach in CellChat to identify common signaling pathway patterns in multiple cell types. CellChat showed that B cells and CD4 cells mainly secreted CD40, IL16, and TNF signals; CD8 mainly secreted chemokines, such as CCL and IL2, and inflammatory signals; and myeloid cells secreted IL1, IL4, and CSF signals. In terms of the input signal pattern, VIM epithelial cells showed certain similarities in input and output patterns of fibroblasts, which highlights the possibility of VIM epithelial cells transforming into fibroblasts during chronic bladder inflammation (**Figure 4E**).

## 4 Discussion

CG is a rare bladder mucosal metaplastic disease that mainly manifests by recurrent frequent urination, dysuria and gross hematuria, eventually leading to hydronephrosis if not treated in time (44, 45). Due to the current lack of research on CG, no standard treatment schedule is available. In this study, single-cell RNA sequencing analysis helped us identify multiple cell subtypes, cell functions, and interactions between cell groups in the immune microenvironment of CG for the first time, which bring light to its pathogenesis and possible treatment approaches at the cellular level.

The conversion of cystitis glandularis into bladder adenocarcinoma has been subjected to extensive debate in the past (4, 46). Here, we found that urothelial cells in CG do not contain the classical copy number variation observed in bladder malignant tumors, raising the possibility that CG and bladder adenocarcinoma may be two separate diseases (47, 48). IgA and IgM secreted by plasma cells need to cross the mucosal epithelial cells to reach the mucosal surface *via* transcytosis, a process that depends on a key receptor protein known as polymeric immunoglobulin receptor (PIGR). PIGR specifically binds secreted IgA and IgM molecules containing J chains on the basal membrane side of epithelial cells, and then transports them to the mucosal side (49, 50). At the same time, accumulating evidence has shown that, in addition to plasma cells, a variety of epithelial cells can secret immunoglobulins (51–53). Moreover, these non-B-Igs not only have specific immune activity, but also promote the growth and migration of various tumor cells (54, 55). In cystitis glandularis, TAO XU et al. detected a strong stain of IgG in both cystitis glandularis patient samples and bladder cancer samples, and the overexpression of IgG can promote abnormal proliferation and migration of bladder urothelial cells. However, in normal bladder mucosa, no significant expression of IgG was found (56). Lennart Enerback believes that in metaplastic lesions of the urinary bladder urothelium, bladder urothelial cells can recruit mast cells through immunoglobulin E-mediated reactions, and the recruited mast cells can further promote the progression of chronic inflammation by releasing histamine and other substances (57). This is consistent with our results, in which the accumulation of mast cells in the microenvironment of CG was observed. These evidences suggest that the overexpression of immunoglobulins may be induced by various pro-cancer or pro-inflammatory factors, and further promote the development of chronic bladder inflammation. Interestingly, we found that PIGR_epithelial cells are intestinal-type glandular cells and enriched in a variety of immunoglobulin-related genes, suggesting an important role for PIGR_epithelial cells in the humoral immune defense of CG.

We then analyzed a variety of immune cells in CG. We demonstrated massive proliferation and strong cytotoxic effect of NK cells in CG. Moreover, T cells are generally inhibited by LAG3 and PDCD1, but are still able to express a variety of chemokines, such as IL-17R and CXCL13, in a dysfunctional state. As a specific transcriptional regulator in Treg cells, FOXP3 plays an important role in inducing the differentiation and immunosuppressive function of Treg cells (58, 59). We confirmed that the IL-2-STAT5 signaling pathway promotes an active state of Treg cells through FOXP3 in CG, inhibiting self-injury caused by excessive inflammation.

B cells are the dominant cell type in adaptive immunity, affecting the activation of T cells and the secretion of major immunoglobulins. Lambrechts et al. also reported the presence of CD27-IGHD+ mature naive cells in the extensive atlas of pan-cancer, and identified CD27- memory B cells (60). In contrast, we found that CD27-SMB simultaneously expressed a variety of mature immunoglobulins in CG, highlighting an immune defense function for CD27-memory B cells. In addition, we demonstrated that antigen-presenting and proliferation of B cells are not significantly linearly correlated, which is in contrast with previous studies (19) and suggests that both initial memory B cells and mature plasma cells induce immune response through different pathways.

Finally, we analyzed the main immune cell populations of innate immunity, and found that while the proportion of macrophages was significantly increased and some macrophages expressed CCL2 in CG, they were more inclined to the functional polarization of anti-inflammatory M2 macrophages. It is suggested that in the process of bladder chronic inflammation, the immune dysfunction state of innate immune cells.

The TNF superfamily of tumor necrosis factors plays important physiological and pathological roles. The dysregulation of TNF-driven inflammatory pathways is a common mechanism leading to immune-mediated inflammatory diseases (IMID) (61). In the inflammatory environment, TNF-α, as the upstream factor of the inflammatory cascade, can induce stromal cells and epithelial cells to express intercellular adhesion factor (ICAM-1), promote the migration of neutrophils and T cells, and stimulate the release of other inflammatory factors such as IL-22 and IL-6 to promote cell proliferation (62). In our study, we found that KRT15 epithelial cells are highly enriched in TNF inflammatory factors, and activate the downstream TNF-TNFRSF1A-NF-κB P65 positive pathway, thereby regulating epithelial cell proliferation and differentiation. This provides a further theoretical basis for the use of TNF-α inhibitors such as adalimumab, Infliximab, Certolizumab Pegol in treating CG. In summary, we identified several cell types differences between normal tissue and glandular hyperplasia tissue in cystitis glandularis using single-cell technology, clarifying the role of multiple cell subtypes in chronic bladder inflammation. Our study significantly advances the research on cystitis glandularis at the cellular level and provides a theoretical basis for the future treatment of cystitis glandularis.

## 5 Conclusion

In conclusion, we identified several cell types differences between normal and inflammatory tissue in cystitis glandularis using scRNA-sequening technology, clarifying the role and signaling networks of various cell subtypes in chronic bladder inflammation. Our study significantly advances the research on cystitis glandularis at the cellular level and provides a theoretical basis for the future treatment of cystitis glandularis.

## Data availability statement

The data presented in the study are deposited in the NCBI SRA repository, accession number PRJNA930968.

## Ethics statement

The studies involving human participants were reviewed and approved by Ethics Committee of Xiangya Hospital of Central South University. The patients/participants provided their written informed consent to participate in this study.

## Author contributions

TZ wrote the article and prepared the figures, HC, YZW, SW, and PD collected the relevant references, YHW collected the clinical specimens, and MC supervised and guided the whole study. All authors contributed to the article and approved the submitted version.
