# Supplementary material for: Single-cell RNA sequencing reveals the immune microenvironment and signaling networks in cystitis glandularis
Source: Front Immunol. 2023 Feb 6;14:1083598. doi: 10.3389/fimmu.2023.1083598 (PMC9940314; doi:10.3389/fimmu.2023.1083598)
Supplement: Supplementary file 1 [file DataSheet_1.pdf]

Supplementary Material

Supplementary Figure S1

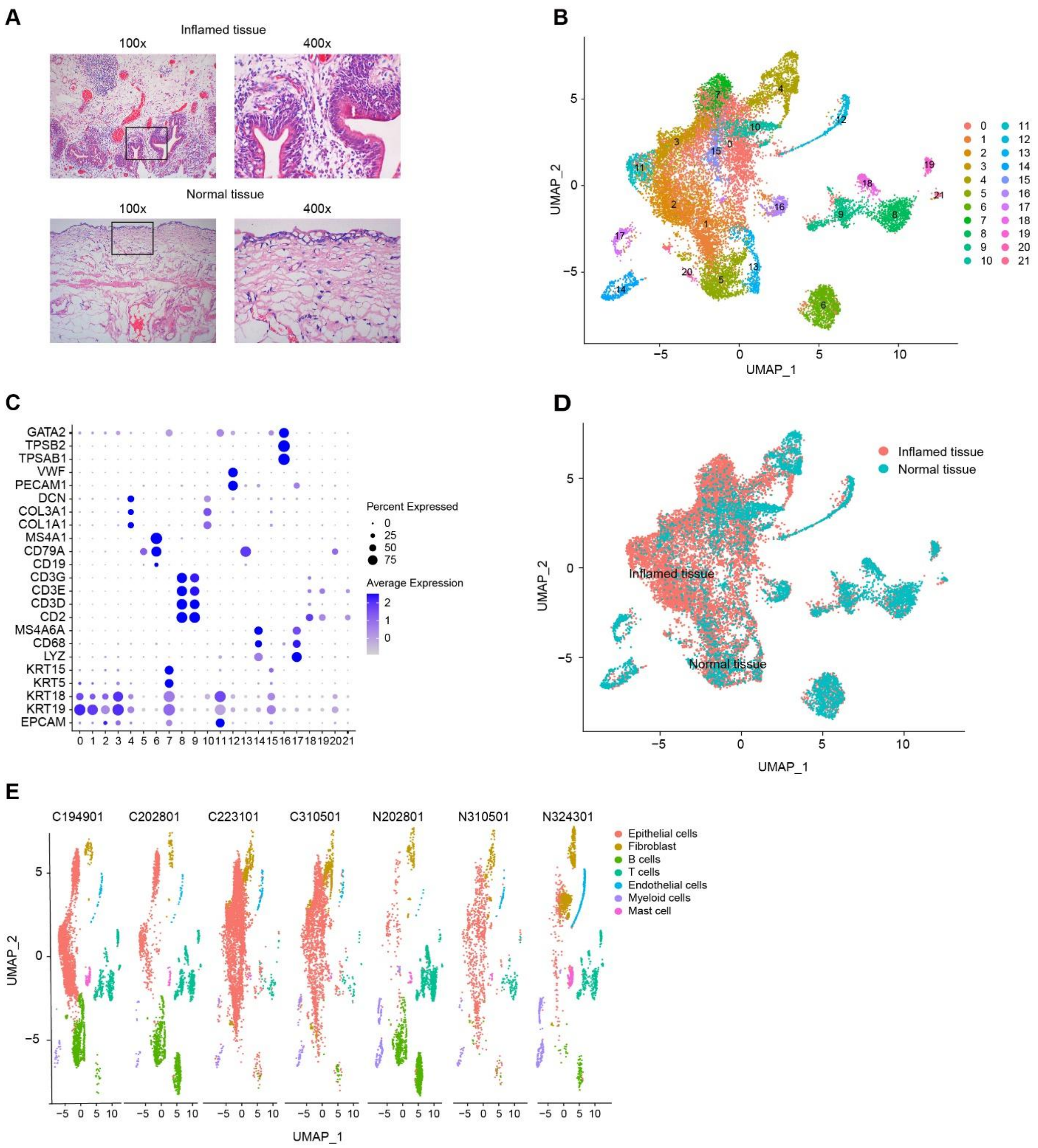

Supplementary Figure S1. Characteristics of various cell types and analysis of epithelial cells in CG (Related to Figure 1). A: HE staining images of inflammatory (above) and surrounding normal tissue (bottom) of CG, 100x field of view at left and 400x field of view at right. B: Reduced dimension visualization U-MAP plot (resolution = 0.5) after cell clustering by Seurat, in which each color represents a cell group. C: Dot plot of canonical genes for each cell group after cell clustering. D-E: U-MAP plot of the scRNA-seq data showing that the evenly distribution of different cell types in each sample after data integration.

Supplementary Figure S2

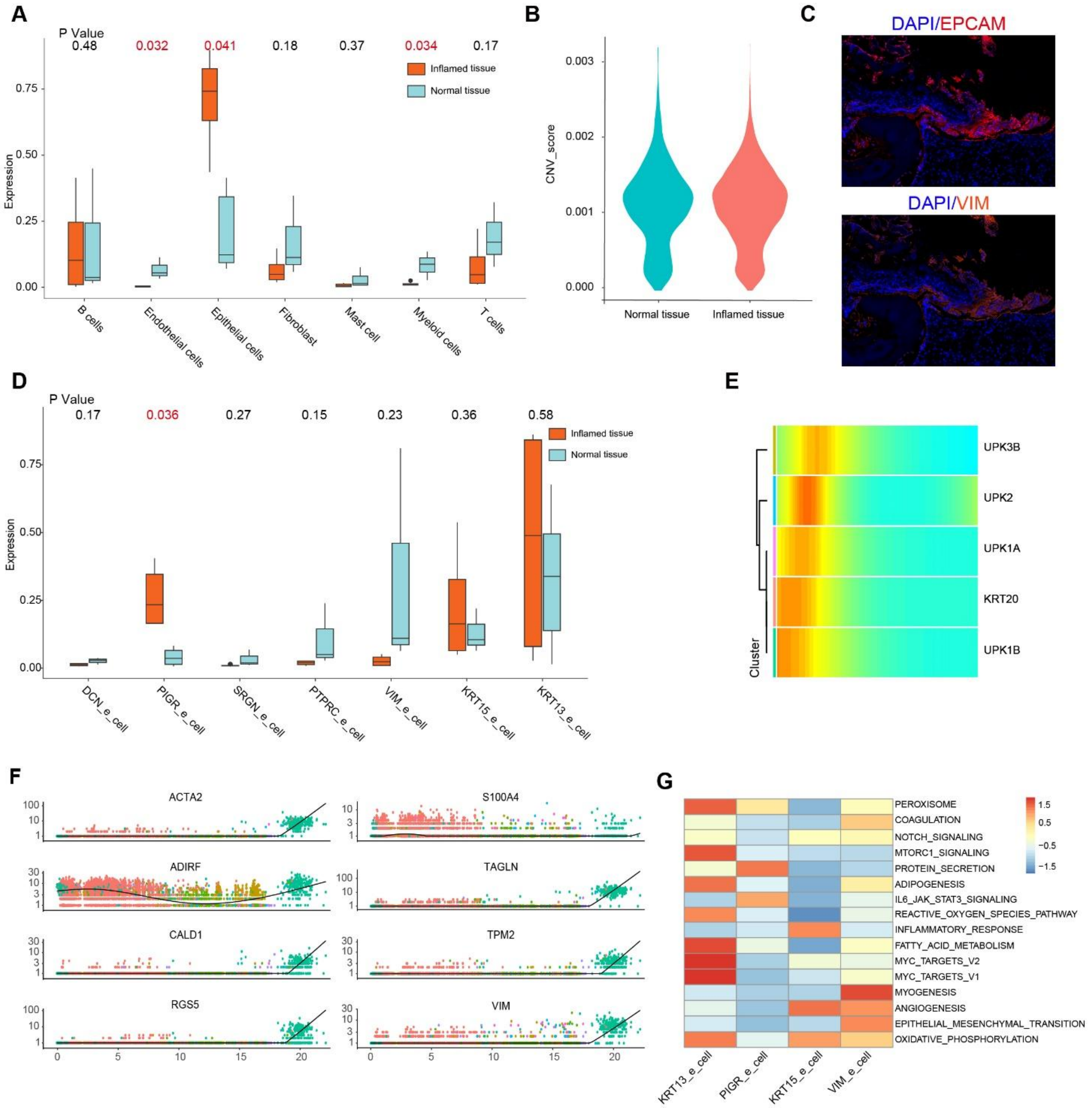

Figure S2. Characteristics of epithelial cell subsets in CG (Related to Figure 1 and Figure 2). A: The comparative analyses of proportions of different cell types CG vs control. The proportion of each cell subset in each patient was calculated, and then Student's t-test was applied to obtain the p value of the comparative analyses CG vs control. B: InferCNV analysis showing copy number variation scores in CG and normal epithelial cells. C: The validation of the existence of VIM epithelial cells by multiple immunofluorescence. D: The comparative analyses of proportions of epithelial cell subsets CG vs control. E-F: Monocle cell trajectory analysis of epithelial cells showing (E) the expression of superficial epithelial cell markers and (F) related protein changes along the differentiation of

epithelial cells. G: Heat map showing the major cellular pathways in epithelial cells conducted by GSVA.

Supplementary Figure S3

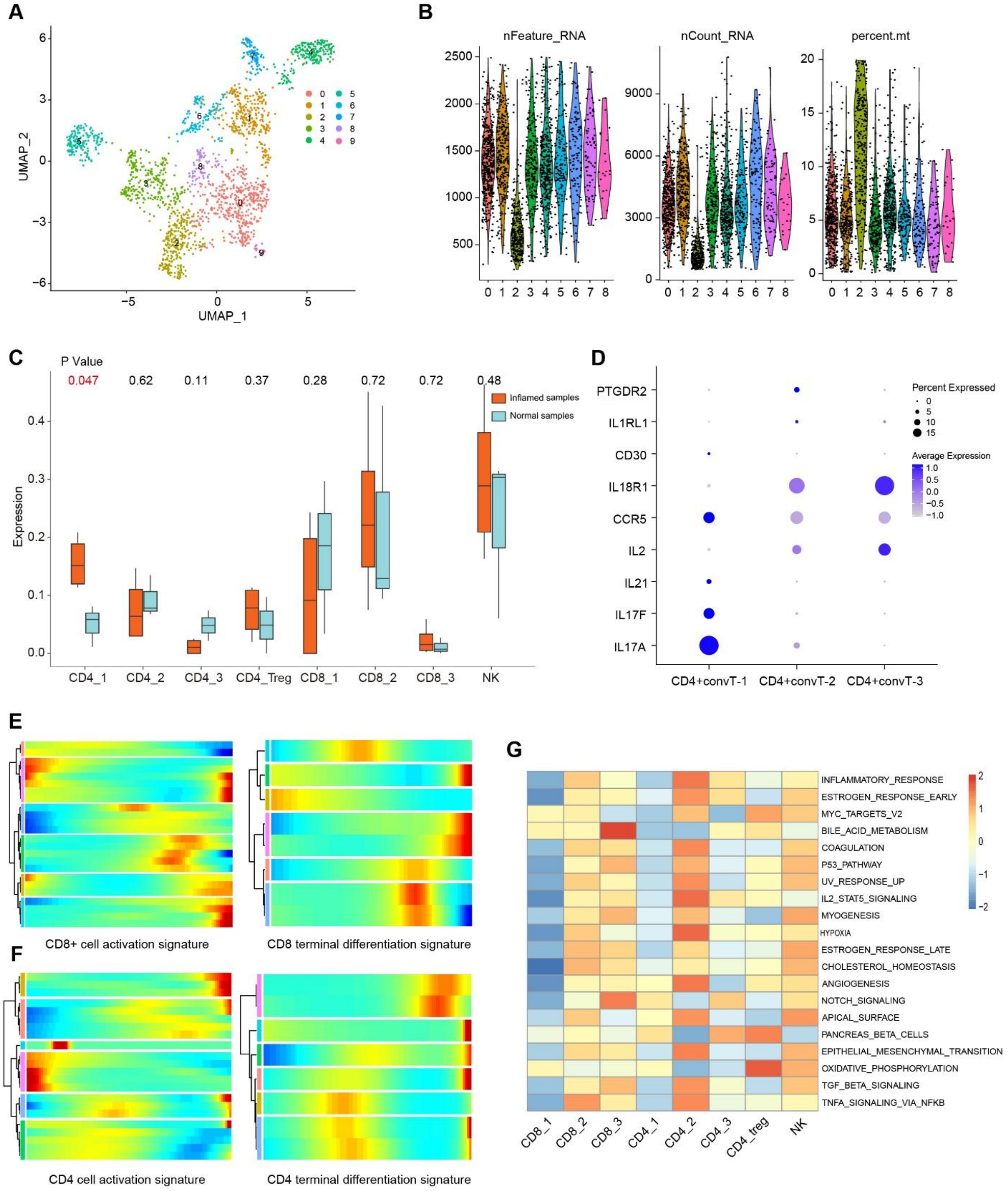

Figure S3. Characteristics of t cell subsets in CG (Related to Figure 3). A: U-MAP plot showing the distribution of T cells after Seurat clustering with a resolution of 0.5. T cells were divided into 9 different cell groups. B: Violin plot showing statistical information for each T-cell subset, including nFeature_RNA (left), nCount_RNA (middle), and percent.mt (right). The deviation of Group_2 cells

was too large, whereby it was excluded from subsequent analyses. C: The comparative analyses of proportions of T cell subsets CG vs control. D: Dot plot of canonical genes for CD4 cell subsets. E-F: Monocle heat map showing the changes of functional genes of CD4+ T cells /CD8+ T cells along the differentiation trajectory. The specific genes can be found in supplementary table S5. G: Heat map displaying the functional pathways of T cell subsets after GSVA enrichment analysis.

Supplementary Figure S4

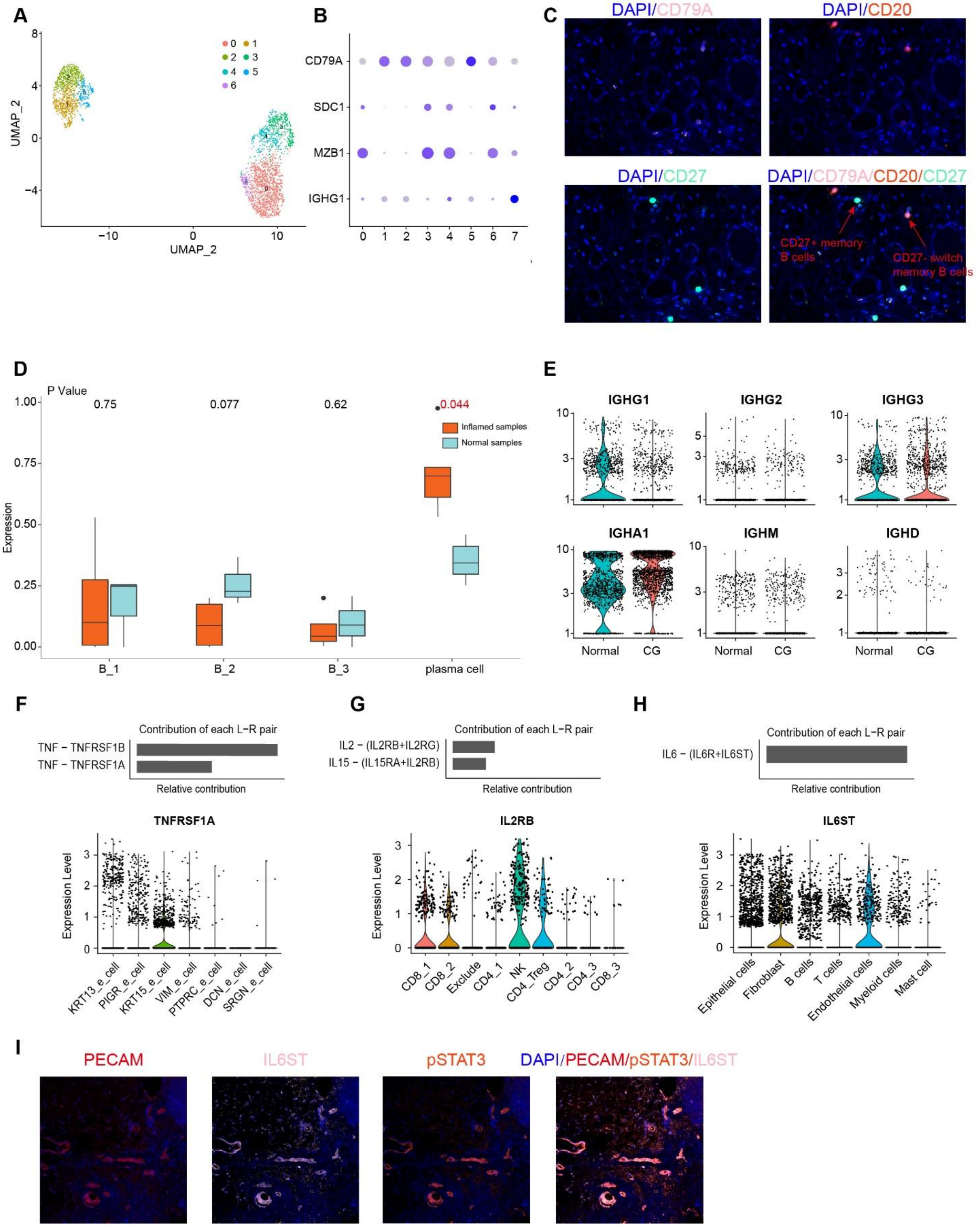

Figure S4. Characteristics of B cell subsets in CG (Related to Figure 4). A: U-MAP showing the two-dimensional distribution of B cell subsets. B: Dot plot showing the canonical gene expression levels of different B cell subsets. C: The validation of the existence of CD27- switched memory B cells by multiple immunofluorescence. D: The comparative analyses of proportions of B cell subsets CG vs control. E: Violin plot showing the expression of immunoglobulin in CG and normal samples. F-H: the strength of specific interactions between receptor and ligand in the inferred cell signal, (top) showing the strength of the recipient ligand relationship in the TNF, IL2, IL6 signaling pathways; and (bottom) the ligand expression in different cell types. I: Multiplex immunofluorescence demonstrating the activation pathway of IL6-pSTAT3 in endothelial cells.
